# Characterisation of North American *Brucella* isolates from marine mammals

**DOI:** 10.1371/journal.pone.0184758

**Published:** 2017-09-21

**Authors:** Adrian M. Whatmore, Claire Dawson, Jakub Muchowski, Lorraine L. Perrett, Emma Stubberfield, Mark Koylass, Geoffrey Foster, Nicholas J. Davison, Christine Quance, Inga F. Sidor, Cara L. Field, Judy St. Leger

**Affiliations:** 1 FAO/WHO Collaborating Centre for Brucellosis, OIE Brucellosis Reference Laboratory, Animal and Plant Health Agency, Addlestone, Surrey, United Kingdom; 2 Scottish Marine Animal Stranding Scheme, SRUC Veterinary Services, Drummondhill, Inverness, United Kingdom; 3 *Mycobacteria* and *Brucella* Section, National Veterinary Services Laboratories, USDA-APHIS, Ames, Iowa, United States of America; 4 Mystic Aquarium & Institute for Exploration, Mystic, CT, United States of America; 5 SeaWorld Parks and Entertainment, San Diego, CA, United States of America; East Carolina University Brody School of Medicine, UNITED STATES

## Abstract

Extension of known ecological niches of *Brucella* has included the description of two novel species from marine mammals. *Brucella pinnipedialis* is associated predominantly with seals, while two major *Brucella ceti* clades, most commonly associated with porpoises or dolphins respectively, have been identified. To date there has been limited characterisation of *Brucella* isolates obtained from marine mammals outside Northern European waters, including North American waters. To address this gap, and extend knowledge of the global population structure and host associations of these *Brucella* species, 61 isolates from marine mammals inhabiting North American waters were subject to molecular and phenotypic characterisation enabling comparison with existing European isolates. The majority of isolates represent genotypes previously described in Europe although novel genotypes were identified in both *B*. *ceti* clades. Harp seals were found to carry *B*. *pinnipedialis* genotypes previously confined to hooded seals among a diverse repertoire of sequence types (STs) associated with this species. For the first time *Brucella* isolates were characterised from beluga whales and found to represent a number of distinct *B*. *pinnipedialis* genotypes. In addition the known host range of ST27 was extended with the identification of this ST from California sea lion samples. Finally the performance of the frequently used diagnostic tool Bruce-ladder, in differentiating *B*. *ceti* and *B*. *pinnipedialis*, was critically assessed based on improved knowledge of the global population structure of *Brucella* associated with marine mammals.

## Introduction

The genus *Brucella* contains an increasing number of species of potentially zoonotic pathogens some of which are responsible for a huge disease, economic and social burden in many parts of the world [1, 2]. Since the first, and unexpected, isolations of *Brucella* from marine mammals [3, 4], it has become clear that *Brucella* are also widespread in the marine ecosystem [5–7] with serological, molecular or cultural evidence for infection from various species of cetaceans and pinnipeds. *Brucella* isolates identified from the marine environment have been shown to be closely related to, but distinct from, species classically associated with terrestrial mammals [8, 9]. Reflecting this they have been formally described as two distinct *Brucella* species, *Brucella ceti*, preferentially associated with cetaceans, and *Brucella pinnipedialis*, preferentially associated with pinnipeds [10].

In recent years substantial numbers of isolates of *Brucella* from marine mammals have been characterised by both phenotypic and molecular approaches particularly multilocus sequence analysis (MLSA) and multilocus variable number of tandem repeat analysis (MLVA) [11–13]. These studies have consistently identified three major groupings of marine mammal *Brucella* isolates–findings are exemplified by studies using a 9 locus MLSA approach (BruMLSA9) that identified five distinct sequence types (STs) corresponding to *Brucella* isolated from marine mammals. While one group corresponds to *B*. *pinnipedialis* (the closely related STs 24 and 25), *B*. *ceti* isolates correspond to two rather distinct groups which appear most commonly associated with dolphins (*Delphinidae)*, and less extensively from beaked whales (*Ziphiidae*) (represented by ST26), or harbour porpoises (represented by ST23). Extension of MLSA studies examining additional strains and using an extended 21 locus approach (BruMLSA21) to increase resolution has further subdivided these groups although the general principle of three major groups has held [14]. For example, isolates specifically from hooded seals belonged to ST25 when examined by BruMLSA9 but represent distinct and unique genotypes (ST53 and ST54) when BruMLSA21 is applied. These findings are consistent with MLVA studies that have shown that isolates from hooded seals represent a distinct grouping [11, 13].

The fifth previously described marine mammal *Brucella* BruMLSA9 genotype, ST27, appears distinct from above groups. ST27 is of particular interest and significance because all three cases of naturally acquired human zoonotic infection assumed to originate from the marine environment [15, 16] were caused by strains of this genotype [17]. However this genotype had, until recently, only been isolated from a bottlenose dolphin (*Tursiops truncatus*) maintained in an open-water management system and thus its natural host and geographical range remain unclear. It had been suggested that this genotype might be preferentially associated with Pacific waters since (i) it had never been represented in isolates from European waters (ii) both human and marine mammal isolates of ST27 characterised to date were associated with Pacific waters, and (iii) further molecular evidence had been reported for infection of a whale in the Pacific with this genotype [17, 18]. However, later evidence pointed to the very widespread presence of this genotype in bottlenose dolphins off the East coast of the USA [19] and there has additionally been a recent report of the presence of ST27 isolated from a single bottlenose dolphin in Europe, from the Adriatic Sea [20].

To date the vast majority of molecular or phenotypic analysis has been confined to isolates from Atlantic or North Sea waters in Northern Europe (largely from waters surrounding the British Isles, Germany or Norway). Recently ST26 isolates were described in striped dolphins (*Stenella coeruleoalba*) in Mediterranean waters [21–23] and isolates that likely correspond to ST26, described on the basis of MLVA, were isolated from striped and a bottlenose dolphin from the Western Mediterranean [24]. However, while serological evidence suggests that *Brucella* infection is widespread globally [5], very limited characterisation of isolates outside Europe has been described. The Americas represent a particular gap in knowledge with significant serological evidence of infection in marine mammals in North and Central American waters [25–28] but no extensive characterisation of isolates described since the initial descriptions of *Brucella* from marine mammals.

To address this gap we undertook a comparative molecular and phenotypic analysis examining a collection of isolates from North American waters (both Pacific and Atlantic) in order to determine the relationships of North American isolates to those previously described in Europe. Of particular interest was determining the natural host and geographical distribution of ST27 isolates associated with zoonotic infection but until recently not detected in European waters, whether North American isolates are identical to, or distinct from, those seen in Europe and whether host/pathogen relationships are distinct from those seen in Europe. We also assess the applicability of current diagnostic approaches to distinguishing *B*. *ceti* and *B*. *pinnipedialis* based on increasing understanding of the relationships within, and between, these species.

## Methods

### Strains

The strain collection examined in this study (n = 61) was assembled at the National Veterinary Services Laboratories, Ames, Iowa via a number of submitting laboratories across USA and Canada (Table 1 and S1 Table). Isolates were stored in tryptic soy broth containing 25% glycerol at -80°C prior to transport to the Animal & Plant Health Agency (APHA), United Kingdom for analysis. Isolates were revived on serum dextrose agar prior to phenotypic examination and preparation of crude lysates [29] or genomic DNA [30] for molecular studies.

**Table 1:** Summary of molecular and phenotypic characteristics of the North American marine mammal *Brucella* panel.

Strain	Species	Host	*omp*	Bruceladder	Bruceladder*	Bruceladder^#^	IRS	BruMLSA21	IS*711*	Urea	H_2_S	CO_2_	BF	TH	A	M	Wb	Tb	BK_2_	Fi	Iz	R/C
			typing	Profile	Mayer-Scholl *et al*	Kang *et al*	PCR	(MLSA9)	fingerprint													
					794/813bp band	766 bp band																
F8/08-32	*B*. *pinnipedialis*	Beluga whale	O(I)	Marine Mammal	Larger (813bp)	Present	PCR I	ST24 (ST24)	Pattern 6	+	-	+	+	+	+	-	CL	NL	CL	NL	PL	NL
F8/08-2	*B*. *pinnipedialis*	Harbor seal	O(I)	Marine Mammal	Larger (813bp)	Present	NEG	ST25 (ST25)	Pattern 3	+/-	-	+	+	+	+	-	NL	NL	PL	NL	PL	NL
F8/08-3	*B*. *pinnipedialis*	Ringed seal	O(I)	Marine Mammal	Larger (813bp)	Present	PCR I	ST25 (ST25)	Pattern 6	+	-	+	+	+	+	-	PL	NL	PL	NL	PL	NL
F8/08-4	*B*. *pinnipedialis*	Harbor seal	O(I)	Marine Mammal	Larger (813bp)	Present	NEG	ST25 (ST25)	Pattern 4	+/-	-	+	+	+	+	-	PL	NL	PL	NL	PL	NL
F8/08-5	*B*. *pinnipedialis*	Ringed seal	O(I)	Marine Mammal	Larger (813bp)	Present	PCR I	ST25 (ST25)	Pattern 6	+	-	+	+	+	+	-	PL	NL	PL	NL	PL	NL
F8/08-6	*B*. *pinnipedialis*	Ringed seal	O(I)	Marine Mammal	Larger (813bp)	Present	NEG	ST25 (ST25)	Pattern 4	+/-	-	+	+	+	+	-	PL	NL	PL	NL	PL	NL
F8/08-7	*B*. *pinnipedialis*	Ringed seal	O(I)	Marine Mammal	Larger (813bp)	Present	PCR I	ST25 (ST25)	Pattern 4	+/-	-	+	+	+	+	-	PL	NL	PL	NL	PL	NL
F8/08-8	*B*. *pinnipedialis*	Ringed seal	NP	Marine Mammal	Larger (813bp)	Present	PCR I	ST25 (ST25)	Pattern 4	+/-	-	+	+	+	+	-	PL	NL	PL	NL	PL	NL
F8/08-9	*B*. *pinnipedialis*	Harp seal	O(I)	Marine Mammal	Larger (813bp)	Present	NEG	ST25 (ST25)	Pattern 4	+	-	+	+	+	+	-	PL	NL	PL	NL	PL	NL
F8/08-18	*B*. *pinnipedialis*	Harp seal	O(I)	Marine Mammal	Larger (813bp)	Present	PCR I	ST25 (ST25)	Pattern 6	+/-	-	+	+	+	+	-	NL	NL	NL	NL	PL	NL
F8/08-19	*B*. *pinnipedialis*	Harbor seal	O(I)	Marine Mammal	Larger (813bp)	Present	PCR I	ST25 (ST25)	Pattern 6	+	-	+	+	+	+	-	NL	NL	NL	NL	NL	NL
F8/08-20	*B*. *pinnipedialis*	Ringed seal	O(I)	Marine Mammal	Larger (813bp)	Present	PCR I	ST25 (ST25)	Pattern 6	+/-	-	+	+	+	+	-	NL	NL	NL	NL	NL	NL
F8/08-21	*B*. *pinnipedialis*	Ringed seal	O(I)	Marine Mammal	Larger (813bp)	Present	PCR I	ST25 (ST25)	Pattern 6	+	-	+	+	+	+	-	PL	NL	PL	NL	CL	NL
F8/08-22	*B*. *pinnipedialis*	Beluga whale	O(I)	Marine Mammal	Larger (813bp)	Present	NEG	ST25 (ST25)	Pattern 4	++	-	+	+	+	+	-	PL	NL	PL	NL	PL	NL
F8/08-25	*B*. *pinnipedialis*	Ringed seal	O(I)	Marine Mammal	Larger (813bp)	Present	PCR I	ST25 (ST25)	Pattern 3	+	-	+	+	+	+	-	CL	NL	CL	NL	CL	NL
F8/08-26	*B*. *pinnipedialis*	Beluga whale	O(I)	Marine Mammal	Larger (813bp)	Present	PCR I	ST25 (ST25)	Pattern 6	+	-	+	+	+	+	-	PL	NL	PL	NL	CL	NL
F8/08-29	*B*. *pinnipedialis*	Sea otter	O(I)	Marine Mammal	Larger (813bp)	Present	PCR I	ST25 (ST25)	Pattern 6	++	-	+	+	+	+	-	PL	NL	PL	NL	PL	NL
F8/08-30	*B*. *pinnipedialis*	Sea otter	O(I)	Marine Mammal	Larger (813bp)	Present	NEG	ST25 (ST25)	Pattern 4	++	-	+	+	+	+	-	NL	NL	PL	NL	NL	NL
F8/08-35	*B*. *pinnipedialis*	Harbor seal	O(I)	Marine Mammal	Larger (813bp)	Present	NEG	ST25 (ST25)	Pattern 3	+	-	+	+	+	+	-	NL	NL	NL	NL	NL	NL
F8/08-36	*B*. *pinnipedialis*	Harbor seal	O(I)	Marine Mammal	Larger (813bp)	Present	NEG	ST25 (ST25)	Pattern 3	+/-	-	+	+	+	+	-	NL	NL	NL	NL	NL	NL
F8/08-51	*B*. *pinnipedialis*	Harbor seal	O(I)	Marine Mammal	Larger (813bp)	Present	NEG	ST25 (ST25)	Pattern 4	+	-	+	+	+	+	-	PL	NL	PL	NL	PL	NL
F8/08-52	*B*. *pinnipedialis*	Harbor seal	O(I)	Marine Mammal	Larger (813bp)	Present	NEG	ST25 (ST25)	Pattern 4	+	-	-	+	+	+	-	CL	NL	PL	NL	CL	NL
F8/08-53	*B*. *pinnipedialis*	Northern fur seal	O(I)	Marine Mammal	Larger (813bp)	Not present	NEG	ST25 (ST25)	Pattern 3	+	-	+	+	+	+	-	NL	NL	PL	NL	NL	NL
F8/08-56	*B*. *pinnipedialis*	Harbor seal	O(I)	Marine Mammal	Larger (813bp)	Present	NEG	ST25 (ST25)	Pattern 3	+	-	+	+	+	+	-	NL	NL	NL	NL	PL	NL
F8/09-1	*B*. *pinnipedialis*	Harbor seal	O(I)	Marine Mammal	Larger (813bp)	Present	NEG	ST25 (ST25)	Pattern 4	+	-	+	+	+	+	-	NL	CL	PL	PL	CL	NL
F8/09-2	*B*. *pinnipedialis*	Harbor seal	O(I)	Marine Mammal	Larger (813bp)	Present	NEG	ST25 (ST25)	Pattern 4	+	-	+	+	+	+	-	NL	CL	PL	NL	PL	NL
F8/08-16	*B*. *pinnipedialis*	Harbor seal	L(I)	Marine Mammal	Larger (813bp)	Present	PCR I	ST25 (ST25)	Pattern 6	+/-	-	+	+	+	+	-	NL	NL	NL	NL	NL	NL
F8/08-17	*B*. *pinnipedialis*	Harbor seal	L(I)	Marine Mammal	Larger (813bp)	Present	PCR I	ST25 (ST25)	Pattern 6	+/-	-	+	+	+	+	-	NL	NL	NL	NL	NL	NL
F8/08-27	*B*. *pinnipedialis*	Harbor seal	L(I)	Marine Mammal	Larger (813bp)	Present	PCR I	ST25 (ST25)	Pattern 6	+	-	+	+	+	+	-	NL	NL	PL	NL	PL	NL
F8/08-28	*B*. *pinnipedialis*	Harbor seal	L(I)	Marine Mammal	Larger (813bp)	Present	PCR I	ST25 (ST25)	Pattern 6	+	-	+	+	+	+	-	NL	NL	NL	NL	NL	NL
F8/08-49	*B*. *pinnipedialis*	Harp seal	L(I)	Marine Mammal	Larger (813bp)	Present	PCR I	ST25 (ST25)	Pattern 6	+	-	-	+	+	+	-	NL	NL	PL	NL	PL	NL
F8/08-12	*B*. *pinnipedialis*	Harbor seal	O(I)	Marine Mammal	Larger (813bp)	Present	NEG	ST52 (ST25)	Pattern 4	+/-	-	+	+	+	+	-	NL	NL	PL	NL	NL	NL
F8/08-15	*B*. *pinnipedialis*	Harbor seal	O(I)	Marine Mammal	Larger (813bp)	Present	NEG	ST52 (ST25)	Pattern 4	+/-	-	+	+	+	+	-	NL	NL	NL	NL	NL	NL
F8/08-33	*B*. *pinnipedialis*	Harbor seal	O(I)	Marine Mammal	Larger (813bp)	Present	NEG	ST52 (ST25)	Pattern 3	++	-	+	+	+	+	-	NL	NL	NL	NL	NL	NL
F8/08-34	*B*. *pinnipedialis*	Harbor seal	O(I)	Marine Mammal	Larger (813bp)	Present	NEG	ST52 (ST25)	Pattern 3	++	-	+	+	+	+	-	NL	NL	PL	NL	PL	NL
F8/08-37	*B*. *pinnipedialis*	Harbor seal	O(I)	Marine Mammal	Larger (813bp)	Present	NEG	ST52 (ST25)	Pattern 3	+	-	+	+	+	+	-	NL	NL	PL	NL	PL	NL
F8/08-38	*B*. *pinnipedialis*	Harbor seal	O(I)	Marine Mammal	Larger (813bp)	Present	NEG	ST52 (ST25)	Pattern 3	+	-	+	+	+	+	-	NL	NL	NL	NL	NL	NL
F8/08-39	*B*. *pinnipedialis*	Harbor seal	O(I)	Marine Mammal	Larger (813bp)	Present	NEG	ST52 (ST25)	Pattern 3	+	-	+	+	+	+	-	NL	NL	NL	NL	NL	NL
F8/08-40	*B*. *pinnipedialis*	Harbor seal	O(I)	Marine Mammal	Larger (813bp)	Present	NEG	ST52 (ST25)	Pattern 3	+	-	+	+	+	+	-	NL	NL	PL	NL	PL	NL
F8/08-41	*B*. *pinnipedialis*	Harbor seal	O(I)	Marine Mammal	Larger (813bp)	Present	NEG	ST52 (ST25)	Pattern 3	+/-	-	+	+	+	+	-	NL	NL	NL	NL	PL	NL
F8/08-42	*B*. *pinnipedialis*	Harbor seal	O(I)	Marine Mammal	Larger (813bp)	Present	NEG	ST52 (ST25)	Pattern 3	+/-	-	+	+	+	+	-	NL	NL	NL	NL	NL	NL
F8/08-43	*B*. *pinnipedialis*	Harbor seal	O(I)	Marine Mammal	Larger (813bp)	Present	NEG	ST52 (ST25)	Pattern 3	+	-	+	+	+	+	-	NL	NL	NL	NL	NL	NL
F8/08-44	*B*. *pinnipedialis*	Harbor seal	O(I)	Marine Mammal	Larger (813bp)	Present	NEG	ST52 (ST25)	Pattern 3	+	-	-	+	+	+	-	NL	NL	PL	NL	NL	NL
F8/08-55	*B*. *pinnipedialis*	Harbor seal	O(I)	Marine Mammal	Larger (813bp)	Present	NEG	ST52 (ST25)	Pattern 3	+	-	+	+	+	+	-	NL	NL	NL	NL	PL	NL
F8/08-57	*B*. *pinnipedialis*	Harbor seal	O(I)	Marine Mammal	Larger (813bp)	Present	NEG	ST52 (ST25)	Pattern 3	+	-	+	+	+	+	-	NL	NL	NL	NL	PL	NL
F8/08-10	*B*. *pinnipedialis*	Harp seal	P(I)	Marine Mammal	Larger (813bp)	Present	NEG	ST53 (ST25)	Pattern 7	+/-	-	+	+	+	+	-	CL	CL	PL	PL	CL	NL
F8/08-13	*B*. *pinnipedialis*	Harp seal	P(I)	Marine Mammal	Larger (813bp)	Present	NEG	ST53 (ST25)	Pattern 7	+	-	+	+	+	+	-	CL	PL	PL	PL	CL	NL
F8/08-14	*B*. *pinnipedialis*	Harp seal	P(I)	Marine Mammal	Larger (813bp)	Present	NEG	ST53 (ST25)	Pattern 7	+	-	+	+	+	+	-	PL	NL	NL	PL	PL	NL
F8/08-11	*B*. *pinnipedialis*	Harp seal	P(I)	Marine Mammal	Larger (813bp)	Present	NEG	ST54 (ST25)	Pattern 7	+	-	+	+	+	+	-	CL	CL	PL	PL	PL	NL
F8/08-50	*B*. *ceti* (dolphin clade)	Bottlenose dolphin	N(K)	Marine Mammal	Larger (813bp)	Present	PCR IV	ST26 (ST26)	Pattern 10	++	-	-	+	+	-	-	CL	NL	CL	PL	CL	NL
F8/08-23	*B*. *ceti* (dolphin clade)	Bottlenose dolphin	R(I)	Marine Mammal	Larger (813bp)	Present	NEG	ST103 (ST61)	Pattern 8	++	-	-	-	+	+	-	CL	NL	CL	PL	CL	NL
F8/08-31	*B*. *ceti* (dolphin clade)	Bottlenose dolphin	R(I)	Marine Mammal	Larger (813bp)	Present	NEG	ST103 (ST61)	Pattern 8	++	-	-	+	+	+	-	CL	CL	CL	CL	CL	NL
F8/08-45	*B*. *ceti* (porpoise clade)	Harp seal	M(J)	Marine Mammal	Smaller (794bp)	Not present	PCR III	ST102 (ST23)	Pattern 9	++	-	-	+	+	+	-	NL	NL	PL	NL	PL	NL
F8/08-46	*B*. *ceti* (porpoise clade)	Harp seal	M(J)	Marine Mammal	Smaller (794bp)	Not present	PCR III	ST102 (ST23)	Pattern 9	++	-	-	+	+	+	-	NL	NL	PL	NL	PL	NL
F8/08-1	*B*. *ceti*	Bottlenose dolphin	Q(I)	NP	Band not present	Present	PCR I	ST27 (ST27)	Pattern 5	+	-	-	+	+	-	+	CL	NL	PL	NL	PL	NL
F8/08-24	*B*. *ceti*	California sea lion	Q(I)	NP	Band not present	Present	PCR I	ST27 (ST27)	Pattern 1	++	-	-	+	+	-	+	CL	NL	PL	NL	PL	NL
F8/08-47	*B*. *ceti*	Bottlenose dolphin	Q(I)	NP	Band not present	Present	PCR I	ST27 (ST27)	Pattern 1	++	-	-	+	+	-	+	PL	NL	PL	NL	NL	NL
F8/08-48	*B*. *ceti*	Bottlenose dolphin	Q(I)	NP	Band not present	Present	PCR I	ST27 (ST27)	Pattern 1	++	-	-	+	+	-	+	PL	NL	PL	NL	NL	NL
F8/08-54	*B*. *ceti*	California sea lion	Q(I)	NP	Band not present	Present	PCR I	ST27 (ST27)	Pattern 1	++	-	-	+	+	-	+	CL	NL	CL	NL	PL	NL
F8/08-58	*B*. *ceti*	California sea lion	Q(I)	NP	Band not present	Present	PCR I	ST27 (ST27)	Pattern 1	++	-	-	+	+	-	+	NL	NL	NL	NL	NL	NL
F8/08-59	*B*. *ceti*	California sea lion	Q(I)	NP	Band not present	Present	PCR I	ST27 (ST27)	Pattern 1	++	-	-	+	+	-	+	NL	NL	PL	NL	NL	NL

Urea = urease production, H_2_S = hydrogen sulphide production, CO_2_ = carbon dioxide requirement, BF = growth on media containing fuchsin, TH = growth on media containing thionin, A and M = agglutination with monospecific antiserum, Phage typing: Wb = Weybridge, Tb = Tbilisi, BK_2_ = Berkeley =, Fi =, Firenze Iz = Izatnagar, R/C = rough strains, CL = confluent lysis, NL = no lysis, PL = partial lysis, NP = New Pattern.

*Scholl *et al*. Bruceladder [32]–Size of discriminatory 794/813bp band.

^#^Kang *et al*. Bruceladder [33]–Presence of 766 bp band suggested as discriminatory between *B*. *pinnipedialis* and *B*. *ceti*.

### Phenotypic analysis

Classical phenotypic typing (biotyping) traditionally applied to separate species and biovars of *Brucella* was carried out according to established protocols [1, 31].

### Genotypic analysis

Tools applied to the strain collection examined here have all been described in detail elsewhere. MLSA examining 21 loci was carried out as described previously [14]. Full MLSA profiles of all marine mammal *Brucella* isolates examined to date at APHA (both European and North American) are given in S2 Table. IS*711* fingerprinting, IRS PCR and *omp* typing were carried out as described in a previous study characterising European isolates of *Brucella* from marine mammals by identical approaches [12]. The Bruce-ladder multiplex PCR assay was carried out using the approach described previously [32]. In addition the performance of a novel primer pair identified in a later study as potentially being useful to differentiate *B*. *ceti* and *B*. *pinnipedialis* (5’CCAACCGTATGTCCTCTCT3’ and 5’TGCGGGAACTGGTGTTCGAC3’) was examined using the published conditions [33].

### Phylogenetic analysis

The phylogenetic tree used in this manuscript was constructed using a representative strain of all BruMLST21 genotypes (sequence types or STs) seen in a recent global study examining the *Brucella* genus population structure [14] plus two additional novel STs identified in this study. Here we present only the extracted subtree corresponding to marine mammal *Brucella* for ease of visualisation. To visualise overarching relationships with other *Brucella* species readers should refer to the topology shown in [14]. Sequences of the 21 loci were concatenated to produce a 10,257 bp sequence (including indels) for each genotype. Phylogenetic analysis was performed with the MEGA software, Version 5.2 [34]. Neighbour joining trees were constructed using the Jukes-Cantor model and the percentage bootstrap confidence levels of internal branches were calculated from 1000 resamplings of the original data.

Minimum spanning trees were constructed in Bionumerics using the categorical coefficient. In the tree STs are represented by circles and the size of the circle is indicative of the number of isolates of that particular type. The colouring inside the circles indicates the proportion of isolates from particular host species. Thick solid lines joining two types denote types differing at a single locus while thinner and thinnest solid lines represent types differing at 2 or 3 loci respectively. Dashed lines represent types differing at 4 or more loci. A maximum neighbour difference of 3 was used to create complexes shown shaded grey.

### Data availability

Databases containing allele descriptions and allelic profiles for both BruMLSA9 [35] and the extended BruMLSA21 data described here, and a corresponding isolate database, are freely available at http://www.pubmlst.org/brucella where data can be interrogated and submissions of new data are encouraged. All BruMLSA21 data that forms the basis of the analyses described in this communication have been deposited in these databases.

## Results

### Placement of isolates by MLSA

Marine mammal strains were initially assigned to 5 BruMLSA9 STs in previous studies [35] which, following both extension of the MLSA scheme to examine 21 loci and the characterization of additional strains of European origin, was increased to 18 STs [14]. These initial MLSA studies included only six strains from North America (see S3 Table), five belonging to BruMLSA9 ST25 (corresponding to BruMLSA21 ST52) and the ST27 isolate believed to be associated with the first US isolations of marine mammal *Brucella* [3, 36].

**Fig 1:**
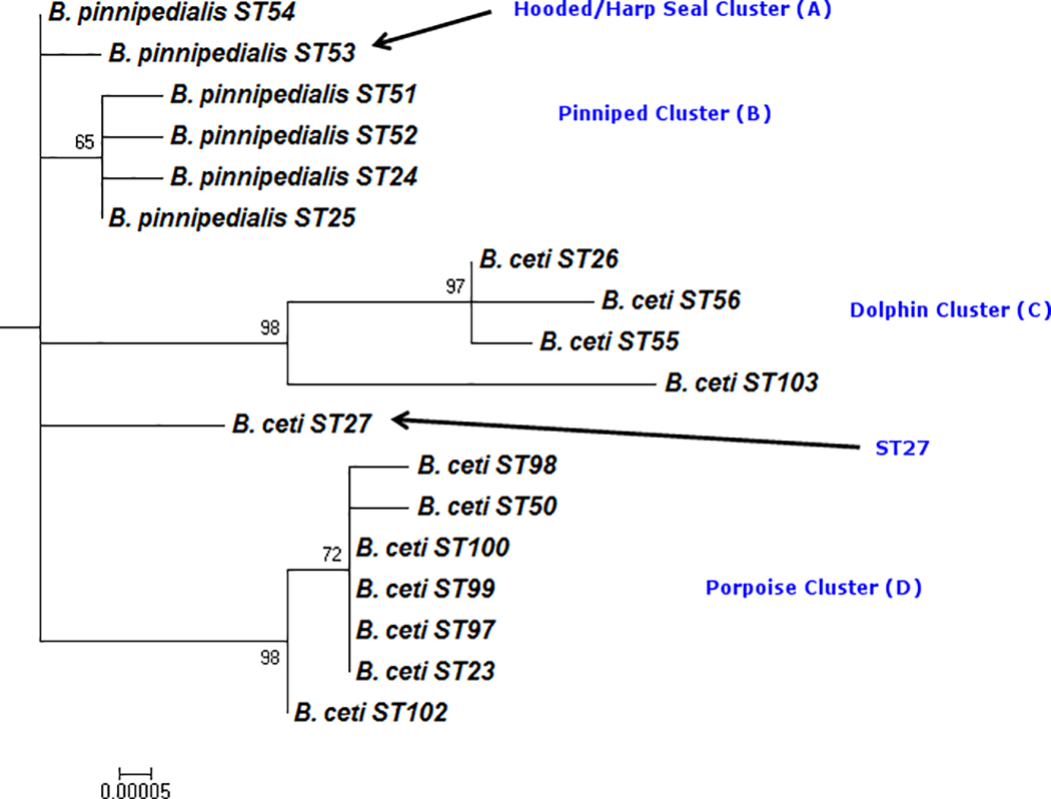
Phylogenetic relationships of BruMLSA21 STs based on concatenated sequence data. Bar = substitutions per nucleotide position.

In this study an additional 61 isolates obtained from North American waters were examined (Table 1 and S1 Table). Of the 61 isolates, 57 represented BruMLSA21 STs also seen in European isolates and reported previously [14] while four isolates were found to represent two distinct novel STs. As shown in Fig 1, which illustrates the phylogenetic placement and host associations of the 61 North American isolates, one novel ST (BruMLSA21 ST103) appears as an early branch in the existing *B*. *ceti* cluster commonly associated with dolphins (Cluster C). This was associated with two bottlenose dolphin isolates from the Texan coastline of the Gulf of Mexico (including one described in a previous case report by Goertz *et al*. [37]). The other, BruMLSA21 ST102, represents a novel lineage within the *B*. *ceti* cluster preferentially associated with porpoises (Cluster D) although the two isolates concerned here came from a single harp seal (*Pagophilus groenlandicus*) from New England.

Of the isolates assigned to previously described genotypes, most belonged to genotypes of the *B*. *pinnipedialis* complex (Cluster B), reflecting the pinniped origin of the majority of the North American isolates examined here. Thirty isolates represented BruMLSA21 ST25 and were derived from harbour (*Phoca vitulina*), ringed (*Pusa hispida***)**, harp and Northern fur seals (*Callorhinus ursinus*) as well as sea otters (*Enhydra lutris*) and beluga whales (*Delphinapterus leucas*). A further 14 isolates, derived solely from harbour seals, represented BruMLSA21 ST52 and a single isolate of BruMLSA21 ST24 was associated with a beluga whale.

In addition to the two novel BruMLSA21 ST103 isolates from bottlenose dolphins falling within the *B*. *ceti* Cluster C, one isolate, obtained from a bottlenose dolphin from the Gulf of Mexico coast, was found to represent the existing genotype BruMLSA21 ST26. Four isolates, all from harp seals in New England, belong to BruMLSA21 ST53 or ST54 (Cluster A), genotypes previously associated exclusively with hooded seals (*Cystophora cristata*) [14]. Finally, BruMLSA21 ST27 was found in three bottlenose dolphin isolates and, for the first time, associated with four isolates originating from California sea lions (*Zalophus californianus*).Two of these cases (F8/08-58 and -59) were from placental tissues sampled during a domoic acid (DA) mortality event described by Goldstein et al. [38]. F8/08-24 was isolated from a stranded animal suffering seizures and presumed domoic acid toxicosis and F8/08-54 from the spleen and lymph nodes of a malnourished and injured stranded female pup.

**Fig 2:**
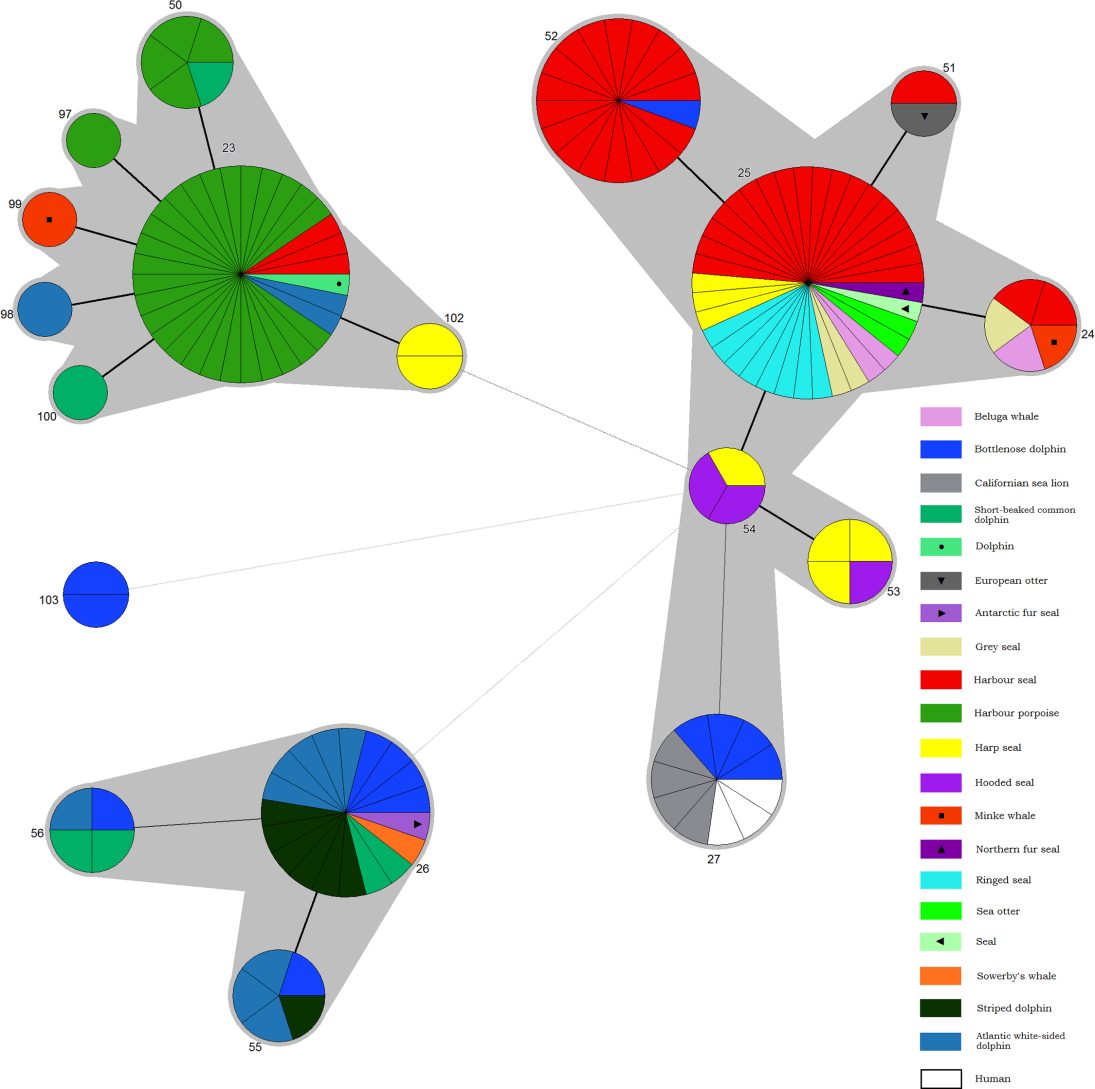
Minimum spanning tree of available BruMLSA21 profiles from European and North American sampling illustrating the relationship between ST and host species. Each circle denotes a particular ST type with the size of the circle illustrating the number of isolates of that particular type. Thick solid lines denote single locus variants (SLV), while thinner and thinnest solid lines represent STs that vary at 2 or 3 loci respectively. Dashed lines types that vary at 4 or more loci. The halos surrounding groupings represent clusters defined in Bionumerics created if neighbours differed in no more than 3 of 21 loci.

Construction of a minimum spanning tree, based on all available BruMLSA21 data (S1 Table), with host species superimposed, confirms the division into three major clonal complexes with distinct preferred hosts of pinnipeds, porpoises and *Delphinidae* and *Ziphiidae* (Fig 2).

### Other molecular typing approaches

As shown below characterization by other molecular typing approaches separated isolates into groupings consistent with those described by BruMLSA21 (see Table 1).

PCR-RFLP of *omp* genes placed isolates of BruMLSA21 ST24, ST25 and ST52 (*B*. *pinnipedialis*) into profiles L(I) or O(I) and isolates of BruMLSA21 ST53 and ST54 (hooded seals) into P(I) consistent with profiles described for European isolates of these clusters previously [12]. With regard to the *B*. *ceti* clade associated with dolphins, the isolate of BruMLSA21 ST26 possessed profile N(K) consistent with some European isolates of this cluster. In contrast the two isolates of the novel BruMLSA21 ST103 possess R(I), a novel *omp* fingerprint profile, consistent with the rather distant relationship with other members of the clade. The two isolates of the novel BruMLSA21 ST102 associated with the *B*. *ceti* cluster preferentially found in porpoises possess profile M(J), consistent with European isolates of this cluster. Finally isolates of BruMLSA21 ST27 all possess *omp* profile Q(I), consistent to that described previously for BruMLSA21 ST27 isolates [12].

The IRS-Derivative PCR examines the presence/absence of four distinct PCR fragments in isolates and, as it was designed based on examination of largely European isolates, its reactivity with the North American isolate panel was examined. Approximately half of the BruMLSA21 ST24, ST25, ST52 (*B*. *pinnipedialis*) group of isolates produced an amplicon with IRS-PCR I as described for European isolates of these genotypes while the remainder did not produce a band with any of the specific PCRs. North American isolates of BruMLSA21 ST53 and BruMLSA21 ST54 do not react with any of the IRS-PCRs in contrast to European hooded seal isolates of these genotypes that react with IRS-PCR I [12]. Isolates of the novel BruMLSA21 ST102 react with IRS-PCR III alone in contrast to European isolates of the *B*. *ceti* clade preferentially associated with porpoises which react with both IRS-PCR II and IRS-PCR III. The isolate of BruMLSA21 ST26 reacted with IRS-PCR IV, as previously described for European isolates of the *B*. *ceti* clade commonly associated with dolphins, however the isolates representing the novel genotype (BruMLSA21 ST103) in this clade produced no reaction with IRS-PCR. Isolates of BruMLSA21 ST27 react with IRS-PCR I as described previously.

Clustering based on IS*711* fingerprinting was also entirely consistent with clustering based on MLSA (S1 Fig) and other molecular approaches. North American isolates corresponding to BruMLSA21 ST24, ST25 and ST52 (MLSA Cluster B–pinniped clade) belonged to one of three closely related fingerprints, isolates of BruMLSA21 ST102 possess another distinct fingerprint (MLSA Cluster D—porpoise clade) and isolates of BruMLSA21 ST53 and BruMLSA21 ST54 another (MLSA Cluster A–hooded/harp seal clade). Again the single isolate of BruMLSA21 ST26 possessed a fingerprint unique among North American isolates while the two isolates of the novel BruMLSA21 ST103 shared a related, but distinct, fingerprint (MLSA Cluster C–dolphin clade). Isolates of BruMLSA21 ST27 possessed one of two distinct fingerprints distinct from other clades.

### Reaction in Bruce-ladder

**Fig 3:**
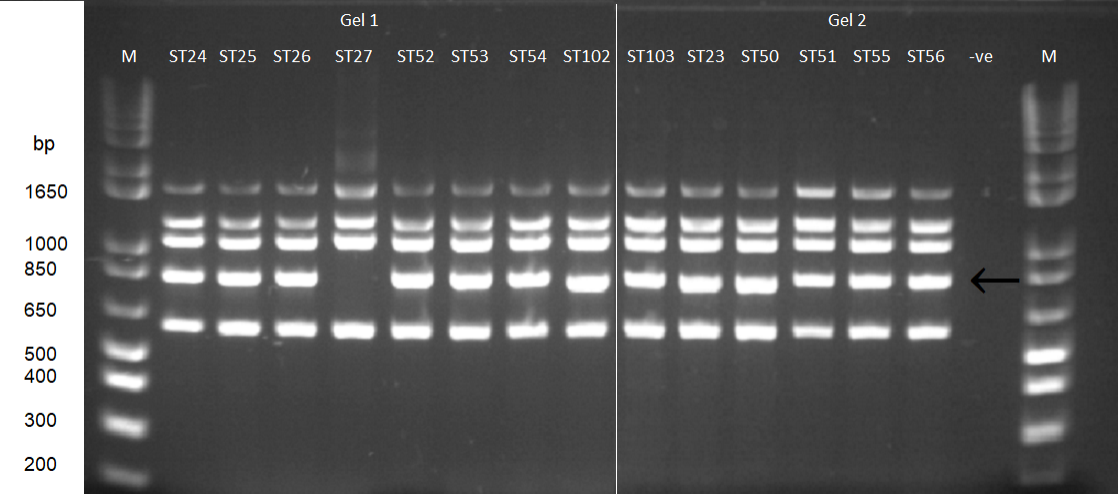
Bruce-ladder profiles of representatives of each of the major marine mammal *Brucella* BruMLSA21 STs based on the Mayer-Scholl protocol [32]. Lane 1. Ladder. Lane 2. ST24 F8/08-32. Lane 3. ST25 F8/08-2. Lane 4. ST26 F8/08-50. Lane 5. ST27 F8/08-1. Lane 6. ST52 F8/08-12. Lane 7. ST53 F8/08-13. Lane 8. ST54 F8/08-11. Lane 9. ST102 F8/08-46. Lane 10. ST103 F8/08-23. Lane 11. ST23 VLA04/72. Lane 12. ST50 UK4/06. Lane 13. ST51 55/94. Lane 14. ST55 UK1/2000. Lane 15. ST56 VLA06/1. Lane 16. PCR negative. Lane 17. Ladder. Arrow = 794bp band.

As the Bruce-ladder multiplex PCR is now a widely used tool for characterization of *Brucella* isolates to species level we examined its performance when applied to the North American strain collection. For a more complete validation we also examined a collection of European isolates representing exemplars of existing STs (S3 Table). It has been reported that a modification of the original Bruce-ladder protocol can differentiate *B*. *ceti* and *B*. *pinnipedialis* isolates [32] based on a small size variation in the 794bp fragment originally described [39]. This fragment is reported to be 19bp shorter in *B*. *ceti* compared to *B*. *pinnipedialis* allowing differentiation of these species, although it is not clear how extensively this difference has been validated. Application of Bruce-ladder revealed that, in our hands, the shorter 794bp fragment appeared confined to the porpoise specific clade of *B*. *ceti* (i.e. BruMLSA21 STs 23, 50, 97–100 and 102). In contrast members of all other STs including all *B*. *pinnipedialis* STs, and STs corresponding to the *B*. *ceti* clade commonly associated with dolphins, possess the larger fragment. Fig 3 shows an example of Bruce-ladder applied to a representative strain of each of the major marine mammal *Brucella* STs. The one exception to these findings was the complete absence of the 794bp fragment in BruMLSA21 ST27 isolates, such that BruMLSA21 ST27 isolates have a Bruce-ladder profile distinct from other isolates of *Brucella* from marine mammals and all other *Brucella* species characterized to date.

An additional modification to Bruce-ladder has been published by Kang *et al*. [33]. They suggested that replacement of the primer set of the 794bp fragment with a novel primer set amplifying a 766bp product might allow discrimination between *B*. *pinnipedialis* and *B*. *ceti* (present only in the former), although they reported testing of only the two species type strains. We therefore tested the performance of this primer set using the same panel of North American and European isolates (Table 1 and S3 Table). Essentially this band was missing only in the *B*. *ceti* porpoise clade being present in *B*. *pinnnipedialis* and the *B*. *ceti* dolphin clade. This is consistent with the reference strain of *B*. *ceti* being a member of the porpoise associated clade.

### Phenotypic analysis

All the North American isolates were subject to classical *Brucella* biotyping. Phenotypic characteristics of marine mammal *Brucella* are known to be rather variable but those of the North American isolates were generally consistent with those previously observed for European isolates. All isolates produce urease but not hydrogen sulphide. *B*. *pinnipedialis* isolates (BruMLSA21 STs 24, 25 and 51–54) grow in the presence of thionine and fuchsin, are serologically A+M- and generally require CO_2_ for growth. The isolates showed limited sensitivity to typing phage with most isolates, like European isolates, resistant to phage Fi and Tb. Sensitivity to phage Bk_2_ and Wb is more variable but, in contrast to European isolates most isolates are also resistant to these phage. Isolates of BruMLSA21 ST102 located in the *B*. *ceti* clade preferentially associated with porpoises showed similar phenotypic profiles to European isolates. Isolates of the *B*. *ceti* clade commonly associated with dolphins generally share the same phenotypic profile again not requiring CO_2_ for growth. Like most European isolates they are largely sensitive to phage Bk_2_, Fi and Wb but resistant to phage Tb. Isolates of BruMLSA21 ST27 showed an atypical profile not requiring CO_2_, growing in presence of thionine and fuchsin but with an A-M+ serological profile. These isolates are resistant to phage Tb and Fi but show variable sensitivity to phage Bk_2_ and Wb.

## Discussion

This paper represents the most extensive phenotypic and molecular characterisation of *Brucella* isolated from marine mammals in North American waters reported to date. Of 61 isolates examined in this study, 57 correspond to genotypes (and corresponding phenotypes) previously seen in Europe but two novel genotypes were seen. One (BruMLSA21 ST102) represents a minor variant of *B*. *ceti* complex preferentially associated with porpoises while the other (BruMLSA21 ST103) represents a more distinct early branch of the *B*. *ceti* complex commonly associated with dolphins. Both of the BruMLSA21 ST103 isolates came from bottlenose dolphins within the Gulf of Mexico. In this region, *Brucella* has been associated with foetal infection and abortion in bottlenose dolphins [40] and comparison of these isolates, to samples associated with abortion, would be of value in indicating any particular association of this novel genotype with pathogenicity. The remaining genotypes have all been reported previously in Northern European waters [11–13].

In general, previous associations of STs with particular host species seen in European isolates were consistent with observations from the North American strains. The North American strain collection is rather biased with the vast majority of isolates being obtained from pinnipeds with only a relatively small number originating from cetaceans. Hence, not unexpectedly, most North American isolates belong to *B*. *pinnipedialis* STs. Around half of the isolates originate from harbour seals with fourteen belonging to BruMLSA21 ST25 previously reported from harbour and grey seals (*Halichoerus grypus*) in Europe and fourteen belonging BruMLSA21 ST52 only previously reported from North American harbour seals. In contrast to BruMLSA21 STs 24 and 25, seen in both Europe and North America, the apparently common ST52 has not been noted in European waters to date. As in Europe, BruMLSA21 ST25 was associated with a range of host species with isolates from ringed seals (8), harp seals (3) a Northern fur seal as well as from sea otters (2) and Beluga whales (2). A single BruMLSA21 ST24 isolate was also found associated with a Beluga whale and to our knowledge these represent the first characterisation of *Brucella* strains associated with this host. It is notable that all three strains associated with these cetaceans represent *B*. *pinnipedialis*.

Isolates from hooded seals have been reported to be distinct from other *B*. *pinnipedialis* isolates on the basis of MLVA analysis [11, 13]. Initially these isolates could not be distinguished from other *B*. *pinnipedialis* (ST25) on the basis of BruMLSA9, although extended 21 locus MLSA does clearly distinguish them into two STs (BruMLSA21 ST53 and BruMLSA21 ST54) only associated with hooded seals to date [14]. Here we extend the known host association of these genotypes as they were also associated with four harp seal isolates from North America (North Atlantic coast of New England). Interestingly, harp and hooded seals share similar habitats and geographical ranges the former being widespread in the Arctic and North Atlantic Oceans and the latter found in the Atlantic region of the Arctic Ocean and high latitudes of the North Atlantic [41].

Of the remaining strains, three bottlenose dolphin isolates were represented by genotypes placed within the *B*. *ceti* complex commonly associated with dolphins. Two represent a novel ST (BruMLSA21 ST103) as already discussed with one isolate representing ST26, a genotype commonly associated with a range of *Delphinidae* and, to a lesser extent, *Ziphiidae* in Europe. Two harp seal isolates from a single animal represent a novel genotype (BruMLSA21 ST102) associated with the *B*. *ceti* complex to date preferentially associated with porpoises. Thus, as reported many times previously for both terrestrial animal and marine mammal associated *Brucella* species, the host associations of particular lineages are strong but not absolute (Fig 2). Some host species in particular, such as harp seals associated with BruMLSA21 STs 25, 102, 53 and 54, appear to harbor a range of *Brucella* genotypes.

One of the principal aims of this study was to attempt to identify the natural host of the genotype BruMLSA21 ST27 until recently only associated with a single case in bottlenose dolphins but with a number of zoonotic human cases [17] thought to be derived from Pacific waters. The study revealed the presence of this genotype in bottlenose dolphins from the US West Coast associated with the original case series [3] as well as the first presence of this genotype in a number of free-ranging California sea lions. Clearly sea lions are highly unlikely to be a source of the human zoonotic infections reported to date. None of the human cases reported any contact with marine mammals [15–17] and additionally the range of the California sea lion is limited compared to observations of the distribution of human cases of infection being confined to coastal waters of the Eastern North Pacific from Central Mexico to British Columbia and around the Galapagos islands [41]. While other sea lion species, such as South American (*Otaria flavescens*) or New Zealand (*Phocarctos hookeri*) sea lions, may match with the geographical distribution of human ST27 cases it seems most likely that BruMLSA21 ST27 is circulating through other marine life that may mediate direct contact with humans. While BruMLSA9 ST27 has still not been reported in Northern European waters where the most extensive surveillance to date has occurred, recent studies, where BruMLSA9 ST27 was found commonly in bottlenose dolphins off South Carolina [19] and in a single animal from the Adriatic Sea in Southern Europe [20] have greatly extended the confirmed range of this genotype.

Although there were no North American porpoise isolates included in this study the molecular data are fully congruent with suggestions that the *B*. *ceti* group should be divided into two groups perhaps retaining *B*. *ceti* for porpoise isolates and reclassifying dolphin isolates as *Brucella delphinii* [11]. A number of studies, including comparative genomic analysis, have suggested previously that *B*. *ceti* is a paraphyletic group [14, 42, 43] and this has impacted on the ability to identify diagnostic markers specific for the species within this group as currently defined [1, 44]. Consistent with this was the observation here that two Bruce-ladder approaches previously reported in the literature could not reliably distinguish between *B*. *ceti* and *B*. *pinnipedialis*. Both approaches found markers tentatively reported specific for *B*. *ceti* to be confined only to the porpoise lineage of *B*. *ceti*. With the Mayer-Scholl *et al*. PCR approach [32] the smaller amplicon is associated with only the *B*. *ceti* porpoise clade (Cluster D) with the *B*. *ceti* dolphin clade (Cluster C) and *B*. *pinnipedialis* isolates (Cluster B) both producing a larger amplicon. Similarly the Kang *et al*. PCR [33] 766 bp band fails to amplify in isolates of the *B*. *ceti* porpoise clade (Cluster D) but is amplified in isolates of both *B*. *pinnipedialis* and the *B*. *ceti* dolphin clade. Two independent WGS based analyses [42, 43], which obviously provide greater resolution than MLSA, have confirmed that of the three main clades *B*. *ceti* (dolphin) is the earliest branching, followed by *B*. *pinnipedialis* and then *B*. *ceti* (porpoise). Because of this branching order any marker that defines both *B*. *ceti* groups is also very likely to be present in *B*. *pinnipedialis*. This means that, although the groups can readily be distinguished by multiplex assays such as MLVA or MLSA, the design of yes/no assays for species differentiation based on specific single markers can be challenging.

The phenotypes within the existing marine mammal *Brucella* species tend to be rather variable as reported previously [12]–this is particularly the case for phage lysis patterns. Comparison with extant data from European isolates [12] revealed that perhaps the most reliable differentiating feature is CO_2_ requirement which tends to split *B*. *ceti* and *B*. *pinnipedialis*, although even here there are exceptions and interpretation of this property can be rather subjective. In terms of the novel clusters, the phenotype of the hooded/harp seal group appears congruent with *B*. *pinnipedialis*. The addition of further isolates gives more confidence in the phenotype associated with BruMLSA21 ST27. In agreement with their genetic isolation from other marine mammal *Brucella* they show a unique phenotypic profile. The lack of requirement for CO_2_ would group this complex with *B*. *ceti* in agreement with their tentative placement in genome based phylogenies [42, 43] but the antigenic profile is very different with these isolates being M dominant in contrast to any other group.

In summary this study describes the first extensive characterisation of isolates of *Brucella* from marine mammals in North America. Comparative analysis with extant data from European isolates provides new insights into our understanding of this emerging group and a framework for understanding the likely complex ecological and host/pathogen relationships. Much more extensive sampling is needed to understand global patterns of genotypes but MLSA offers the ideal tool to build such a database. The majority of isolates in this study were found to represent genotypes previously described in Europe, however, novel genotypes were identified in both *B*. *ceti* clades. Harp seals were found to carry the *B*. *pinnipedialis* genotype previously thought to be confined to hooded seals among a diverse repertoire of STs associated with this species. For the first time, *Brucella* isolates were characterised from beluga whales and found to represent a number of distinct *B*. *pinnipedialis* STs. The known host range of the potentially significant ST27 associated with human zoonotic transmission was extended from bottlenose dolphins with the identification of this ST from a number of California sea lion samples. Finally this study included the most comprehensive study to date of the performance of Bruce-ladder in differentiation of marine mammal *Brucella*. Data add to the growing body of evidence regarding the inconsistency of current marine mammal *Brucella* taxonomic groupings with genetic relationships and illustrate how this can hinder the ability to identify diagnostic markers that accurately reflect current nomenclature.

## Supporting information

S1 Table Additional data relating to geographical origin, strain sources, any history and alternative names of the panel of 61 strains described in Table 1. (XLS)

S2 Table Complete BruMLSA9 and BruMLSA21 profiles of 154 isolates of *Brucella* from marine mammals. The North American panel specifically described in this study are highlighted in pink.
(XLS)

S3 Table Additional information on North American strains previously characterised [35] and the strain panel of representative European derived isolates used for extended testing of Bruce-ladder. (XLS)

S1 Fig Representation of the ten IS*711* fingerprints seen in Northern American marine mammal *Brucella* isolates. Profiles were analysed using Bionumerics (Version 6.6, Applied Maths) using the following tolerance settings: optimisation 0%, and position tolerance 1%. Profiles were clustered using the Jaccard coefficient and the UPGMA approach.
(TIF)
