# Supplementary figures and images for: Characterisation of North American Brucella isolates from marine mammals
Source: PLoS One. 2017 Sep 21;12(9):e0184758. doi: 10.1371/journal.pone.0184758 (PMC5608248; doi:10.1371/journal.pone.0184758)

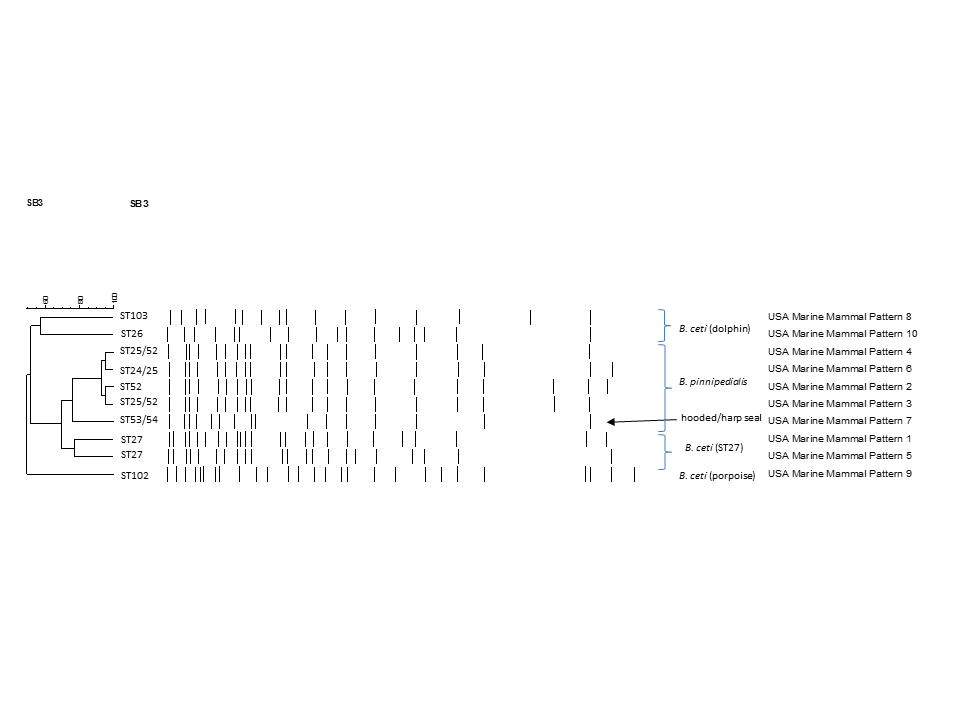

Supplement: S1 Fig — Profiles were analysed using Bionumerics (Version 6.6, Applied Maths) using the following tolerance settings: optimisation 0%, and position tolerance 1%. Profiles were clustered using the Jaccard coefficient and the UPGMA approach. (TIF) [file pone.0184758.s004.tif]
